# Prenatal diagnosis of 1q21.1 microdeletions and microduplications: a retrospective case series

**DOI:** 10.1186/s13039-025-00731-6

**Published:** 2025-09-30

**Authors:** Ziyang Liu, Song Yi, Manman Li, Nian Liu

**Affiliations:** 1 College of Agronomy and Biotechnology, Southwest University, Chongqing, 400716 China; 2 Medical Genetic Center, Maternal and Child Health Hospital of Hubei Province, Tongji Medical College, Huazhong University of Science and Technology, 745 Wuluo Road, Wuhan, 430070 China

**Keywords:** 1q21.1 microdeletion, 1q21.1 microduplication, Prenatal diagnosis, Copy number variation (CNV), Incomplete penetrance, Genetic counseling

## Abstract

**Objective:**

This study aimed to characterize the prenatal features, inheritance patterns, and outcomes of 1q21.1 copy number variations (CNVs) and refine prenatal counseling strategies.

**Methods:**

We conducted a retrospective analysis of 20 consecutive prenatal cases diagnosed with 1q21.1 CNVs between 2017 and 2024. Genetic testing included karyotyping, chromosomal microarray analysis (CMA), and copy number variation sequencing (CNV-seq). Phenotypic data, inheritance patterns, and pregnancy outcomes were evaluated.

**Results:**

The cohort comprised 12 microdeletions (60%) and 8 microduplications (40%). Ultrasound anomalies were detected in 66.7% (8/12) of microdeletion cases (e.g., fetal growth restriction, cardiac defects) and 37.5% (3/8) of microduplication cases (e.g., nasal bone hypoplasia). No specific prenatal ultrasound markers pathognomonic for 1q21.1 CNVs were identified. Termination of pregnancy (TOP) was elected in 50% (10/20) of cases, predominantly for de novo CNVs (80% of TOP decisions). Among live-born infants (*n* = 9), no overt abnormalities were detected during postnatal follow-up (3 months to 5 years).

**Conclusion:**

Prenatal 1q21.1 CNVs demonstrate incomplete penetrance and variable expressivity, without consistent association with specific prenatal ultrasound markers. The TOP rate for de novo CNVs reflects profound parental anxiety regarding neurodevelopmental outcomes. These findings underscore the critical need for comprehensive prenatal counseling that integrates genomic findings, phenotypic severity, and psychosocial support.

## Introduction

Chromosomal copy number variations (CNVs) at the 1q21.1 locus are among the most well-characterized recurrent genomic rearrangements in humans, driven primarily by non-allelic homologous recombination (NAHR) between clustered low- copy repeats (LCRs) in the region [1]. This region (chr1:145.4-147.9 Mb, GRCh37/hg19) demonstrates particularly wide phenotypic variability, ranging from severe neurodevelopmental disorders to asymptomatic presentations [2–4]. Postnatally, affected individuals often present with neurodevelopmental disorders (global developmental delay, intellectual disability, autism spectrum disorder), craniofacial anomalies (microcephaly/macrocephaly), congenital heart defects, and growth disturbances [1, 4–6]. Prenatally, these CNVs present unique diagnostic challenges, with only ~ 50–60%% of cases show detectable ultrasound anomalies [3, 7, 8].

The clinical significance of 1q21.1 CNVs stems from dosage-sensitive genes within the region, particularly *GJA5* (cardiac conduction) and *CHD1L* (neural development) [9, 10]. Despite growing postnatal data, prenatal characterization of 1q21.1 CNVs remains limited, with fewer than 100 reported cases worldwide [3, 7, 8, 11]. This study analyzes 20 consecutive prenatal cases of 1q21.1 CNVs. The indication for prenatal testing, prenatal ultrasound features, inheritance of the CNVs, and the pregnancy outcome were studied. The aim of this study was to comprehensively review prenatal cases of 1q21.1 CNVs to improve understanding of this submicroscopic imbalance in prenatal diagnosis.

## Materials and methods

### Study design and population

A retrospective analysis was conducted at the Prenatal Diagnosis Center of Maternal and Child Health Hospital of Hubei Province (Wuhan, China). Twenty consecutive prenatal cases diagnosed with 1q21.1 CNVs between 2017 and 2024 were included. Written informed consent was obtained from all participants, and the study was approved by the hospital’s Ethics Committee. Detailed information on these cases was extracted from the relevant medical records.

### Genetic testing

1. **Karyotyping**

Amniotic fluid (*n* = 19) or parental peripheral blood samples (*n* = 2) were obtained under sterile conditions. Giemsa staining was performed on metaphase chromosomes from cultured prenatal samples or peripheral blood using established methods, and the resolution of the haploid band was determined with an acceptable range of 320–400.

2. **Chromosome microarray analysis (CMA)**

Genomic DNA was extracted from uncultured amniocytes (*n* = 10) or parental peripheral blood (*n* = 8 couple) using the QIAamp DNA mini-kit (QIAGEN, Germany). DNA quality was assessed using the NanoDrop kit (Thermo Scientific; Waltham, MA, USA). The SNP array analysis was performed using the CytoScan^®^750 K Array (Affymetrix Inc, CA, USA). Genomic DNA labeling and hybridization were performed according to the manufacturer’s protocol. Data were interpreted via Chromosome Analysis Suite (v3.3) against GRCh37/hg19.

3. **Copy number variation sequencing (CNVseq)**

Copy number variation sequencing (CNVseq) was performed on uncultured amniocytes (*n* = 10) or parental peripheral blood (*n* = 8 couple) to detect chromosome anomalies at the low coverage whole-genome sequencing level. Genomic DNA (10 ng) was fragmented, and a DNA library was constructed as previously described [12, 13]. Multiple libraries were indexed, pooled in a single lane, and sequenced on the Nextseq CN500 instrument (Illumina, Inc.) to generate approximately 5 million 45-bp single end reads (including the 8-bp index sequence), resulting in an average sequencing coverage of ~ 0.2×. Genomic imbalances were reported using the GRCh37/hg19 reference genome.

## Results

Over an 8-year period (2017–2024), 20 consecutive prenatal cases (from 19 families) with 1q21.1 CNVs were identified at our center, including 12 microdeletions (60%) and 8 microduplications (40%). Maternal age ranged from 23 to 42 years (mean 32 ± 5.1), with a median gestational age at diagnosis of 20 weeks (17–29 weeks). The main indications for invasive testing were: high-risk maternal serum screening or non-invasive prenatal testing (NIPT) (40%, 8/20), ultrasound anomalies (35%, 7/20) and advanced maternal age (AMA) ≥ 35 years (25%, 5/20). Microdeletions ranged from 0.516 to 2.6 Mb (median 1.9 Mb), and microduplications ranged from 1.28 to 2.5 Mb (median 2.0 Mb) (Table 1).

**Table 1:** Clinical and molecular characteristics of 20 prenatal cases with 1q21.1 CNVs

Case	Maternal Age (y)	Pregnancy history	GA at diagnosis (weeks)	Indication for testing	Ultrasound findings	Sex	Karyotype	Cytogenetic method	Genetic results	CNV size (Mb)	Inheritance	Outcome	
1	29	G2P1	25	High-risk NIPT (Trisomy 16)	None	M	46,XY	CMA	arr[hg19]1q21.1(145372551–145888926)x1	0.516	De novo	TOP	
2	27	G4P1	20	High-risk NIPT (1q21 deletion)	FGR	M	46,XY	CMA	arr[hg19]1q21.1q21.2(146101790–147830830)x1	1.73	Undetermined	TOP	
3	31	G2P1	23	High-risk serum screening (T21)	Polyhydramnios, short femur	M	46,XY	CMA	arr[GRCh37] 1q21.1q21.2(145382123–147830830)x1	2.5	De novo	TOP	
4	31	G2P0	18	Ultrasound anomalies	NT3.1 mm	F	46,XX	CMA	arr[GRCh37] 1q21.1q21.2(145895746–147898062)x1	2	De novo	TOP	
5	33	G2P0	17	Maternal inversion (10)(p13q26)	None	M	46,XY, inv(10)(p13q26)mat	CMA	arr[GRCh37]1q21.1q21.2(145895747–147830830)x1	1.9	Maternal	Live birth	
6	36	G6P2	18	AMA, adverse fertility history	None	M	46,XY	CNV-seq	seq[hg19]1q21.1q21.2(145380000–147400000)x1	2.02	Maternal	Spontaneous abortion at 20 weeks GA	
7	37	G3P1	19	AMA	VSD	F	46,XX	CNV-seq	seq[hg19]1q21.1q21.2(145740000–147840000)x1	2.1	De novo	TOP	
8	34	G3P1	29	Ultrasound anomalies	FGR, short femur	M	N/A	CNV-seq	seq[hg19]1q21.1q21.2(145740000–147840000)x1	2.1	Paternal	TOP	
9	35	G4P2	17	Previous affected pregnancy	None	M	46,XY	CNV-seq	seq[hg19]1q21.1q21.2(145740000–147840000)x1	2.1	Paternal	Live birth	
10	36	G2P1	24	Ultrasound anomalies	PRUV, right subclavian artery anomaly	M	46,XY	CMA	arr[GRCh37]1q21.1q21.2(145895747–147830830)x1	1.9	Undetermined	Live birth	
11	28	G1P0	25	Ultrasound anomalies	Unilateral ventriculomegaly (11 mm)	F	46,XX	CNV-seq	seq[hg19]1q21.1q21.2(146560000–147840000)x1	1.28	De novo	TOP	
12	33	G1P0	19	High-risk serum screening (T21)	Choroid plexus cyst	M	46,XY	CNV-seq	seq[hg19]1q21.1q21.2(145740000–147840000)x1	2.1	Maternal	Live birth	
13	42	G2P1	18	AMA	None	F	46,XX	CMA	arr[GRCh37]1q21.1q21.2(146506904–147391923)x3	0.885	Paternal	Live birth	
14	35	G2P1	18	AMA, ultrasound anomalies	Hypoplastic nasal bone	F	46,XX	CMA	arr[GRCh37]1q21.1q21.2(145382123–147995251)x3	2.6	Maternal	Live birth	
15	23	G1P0	27	Ultrasound anomalies	Unilateral ventriculomegaly (13 mm)	M	46,XY	CMA	arr[GRCh37]1q21.1q21.2(145895747–147856007)x3	1.96	Maternal	Live birth	
16	30	G1P0	17	Ultrasound anomalies	Hypoplastic nasal bone	M	46,XY	CMA	arr[GRCh37]1q21.1q21.2(145390102–147844778)x3	2.5	Paternal	Live birth	
17	30	G1P0	19	High-risk serum screening (T21)	None	M	45,XY, rob(13;14)(q10;q10)	CNV-seq	seq[hg19]1q21.1q21.2)(146500000–147840000)x3	1.34	Undetermined	Live birth	
18	38	G2P1	23	AMA	None	M	46,XY	CNV-seq	seq[hg19]1q21.1q21.2(146500000–147840000)x3	1.34	De novo	TOP	
19	27	G2P1	19	High-risk serum screening (T21)	None	F	46,XX	CNV-seq	seq[hg19]1q21.1q21.2(146500000–147840000)x3	1.34	De novo	TOP	
20	31	G4P2	20	Adverse fertility history	None	M	46,XY	CNV-seq	seq[hg19]1q21.1q21.2(146500000–147840000)x3	1.34	De novo	TOP	

GA: gestational age; NIPT: noninvasive prenatal testing; FGR: fetal growth restriction (estimated fetal weight < 10th percentile); VSD: ventricular septal defect; NT: nuchal translucency; PRUV: persistent right umbilical vein; AMA: advanced maternal age (≥ 35 years); TOP: termination of pregnancy

Ultrasound anomalies were detected in 55% (11/20) of cases (Table 1). In the deletion group (8/12, 66.7%), the anomalies included two cases of fetal growth restriction (FGR), two cases of short femur, one case of ventricular septal defect (VSD), one case of persistent right umbilical vein accompanied by aberrant right subclavian artery (ARSA), one case of ventriculomegaly, one case of increased nuchal translucency (NT), and one case of choroid plexus cyst. In the duplication group (3/8, 37.5%), anomalies consisted of two cases of hypoplastic nasal bone and one case of ventriculomegaly.

Parental test results were available for 17 cases: the origin was de novo in 8 cases and inherited in 9 cases (from 8 families). Termination of pregnancy (TOP) was elected in 50% (10/20) of cases, predominantly for de novo CNVs (8/10). One case resulted in spontaneous abortion at 20 weeks’ gestation. The remaining 9 cases resulted in live births, with no overt abnormalities detected during postnatal follow-up (range: 3 months to 5 years). Detailed clinical and molecular characteristics of the 20 cases are summarized in Table 1.

## Discussion

Our expanded cohort of 20 cases confirms and extends previous findings regarding the prenatal presentation of 1q21.1 CNVs. The 50% detection rate of ultrasound anomalies (10/20 cases) aligns with prior reports of variable expressivity in this region [5, 7]. This variability is not only a challenge for prenatal diagnosis but also indicates the complex nature of 1q21.1 CNVs, which involve multiple genes with diverse functions.

Several studies have associated FGR or short stature with 1q21.1 CNVs [5, 14, 15], echoing the role of *CHD1L* in developmental processes. *CHD1L* encodes a multifunctional protein critical for chromatin remodeling, DNA replication, and cell differentiation, with its highest expression localized to the brain and placental tissues [10, 16]. Specifically, *CHD1L* haploinsufficiency disrupts normal neuronal differentiation and placental development through chromatin remodeling pathways [17], ultimately leading to growth disturbances such as short stature, FGR, and organogenesis defects [4, 5]. This mechanistic link reinforces that FGR in 1q21 CNVs cases is likely a consequence of *CHD1L* dosage loss, rather than incidental comorbidity. As reported in the literature, the disruption of *CHD1L*’s function can lead to a cascade of events that affect placental function and fetal growth, highlighting the importance of this gene in normal development.

Ventriculomegaly observed in 1q21 CNVs also linked to *CHD1L.* Given *CHD1L*’s highest expression in the brain, where it is pivotal for nervous system development, both *CHD1L* haploinsufficiency and overexpression can disrupt cortical development and cerebrospinal fluid flow, leading to ventricular dilation [16, 18]. This further emphasizes the role of *CHD1L* in Maintaining normal brain development and the consequences of its dysregulation in 1q21.1 CNV cases.

*GJA5* encodes connexin 40, a gap junction protein essential for cardiac conduction and septal morphogenesis; deletions of *GJA5* disrupt intercellular communication in developing heart tissue, increasing risk of congenital heart defects (CHDs) like VSD. This aligns with previous finding that 1q21.1 rearrangements are overrepresented in CHD patients [3, 5, 9, 16, 19]. Recent research has shown that CNVs contribute to as Many as 10–15% of CHD cases, with 1q21.1 being one of the genomic hotspots recurrently enriched in CHD cohorts [20]. The specific role of *GJA5* in cardiac development Makes it a key gene in understanding the association between 1q21.1 CNVs and CHDs, and our findings further support this established relationship.

The absence of ultrasound anomalies in nearly half of our cases underscores the diagnostic challenge posed by this CNV’s variable expressivity, as many manifestations only emerge during postnatal neurodevelopment [6, 21]. This is consistent with the understanding that 1q21.1 CNVs often have reduced penetrance and pleiotropic effects on brain and heart development [20]. The complex genetic architecture of these CNVs, along with potential environmental and epigenetic factors, can lead to a wide range of phenotypes that may not be apparent prenatally.

De novo CNVs were strongly associated with pregnancy termination, accounting for 80% of TOP decisions—markedly higher than the 11.1% TOP rate observed in inherited 1q21.1 CNV cases (1/9 inherited cases) in our cohort. This finding aligns with studies demonstrating that de novo CNVs are perceived as higher-risk variants due to their unpredictable neurodevelopmental outcomes [22, 23]. Parental anxiety regarding potential disabilities, coupled with limited prognostic data, likely contributed to the TOP rate. In addition, the incomplete penetrance and variable expressivity of 1q21.1 CNVs, as seen in cases where the same CNV can lead to different outcomes within a family, add to the uncertainty. These observations emphasize the need for improved prenatal counseling protocols integrating genomic data, phenotypic severity, and psychosocial factors to support more informed decision making.

Our findings underscore the complexities of inherited CNVs and their variable phenotypic expression, particularly highlighted by Cases 8 and 9. The siblings inherited the same 2.1 Mb deletion from a phenotypically normal father but exhibited different prenatal outcomes. Case 8 was referred for prenatal diagnosis at 28 weeks’ gestation due to FGR and short femur, leading to TOP after genetic counseling. Case 9—a subsequent pregnancy in the same family—demonstrated normal ultrasound findings and resulted in a healthy term birth. This prenatal phenotypic discordance within a single family exemplifies the incomplete penetrance characteristic of 1q21.1 CNVs, consistent with recent studies reporting 60–70% incomplete penetrance for neurodevelopmental phenotypes [5, 21, 22]. Notably, parents who carried the same 1q21.1 CNV (*n* = 9 cases) underwent genetic testing to confirm inheritance but did not receive formal cognitive assessments or neurodevelopmental evaluations. Thus, we cannot rule out subtle cognitive impairment in these parents—a limitation that should be addressed in future studies to further clarify the phenotypic spectrum of inherited 1q21.1 CNVs.

Our study is limited by its retrospective methodology and modest sample size. Although we did not identify any specific prenatal signs of 1q21.1 CNVs, our findings are consistent with prior reports and comprehensive molecular characterization. Future studies should incorporate long-term neurodevelopmental follow-up and functional analyses of candidate genes (e.g., *GJA5*, *CHD1L*) to refine genotype-phenotype correlations. Emerging evidence implicates calcium signaling dysregulation in 1q21.1-associated neuropsychiatric disorders [23], offering a potential mechanistic framework for future research. Additionally, larger cohort studies are needed to better understand the full spectrum of phenotypes associated with 1q21.1 CNVs, as well as the factors that contribute to their variable expressivity, such as genetic modifiers, epigenetic changes, and environmental exposures. This will not only improve our understanding of the underlying biology but also enhance prenatal diagnostic accuracy and counseling for families affected by these CNVs.

## Data Availability

No datasets were generated or analysed during the current study.
